# Iron nanoparticles to recover a co-contaminated soil with Cr and PCBs

**DOI:** 10.1038/s41598-022-07558-w

**Published:** 2022-03-03

**Authors:** M. Gil-Díaz, R. A. Pérez, J. Alonso, E. Miguel, S. Diez-Pascual, M. C. Lobo

**Affiliations:** 1 Dpto. Investigación Agroambiental, IMIDRA, Finca “El Encín”, A-2, km 38.2, Alcalá de Henares, 28805 Madrid, Spain; 2 Dpto. Medio Ambiente y Agronomía, INIA-CSIC, A-6, km 7, 28040 Madrid, Spain

**Keywords:** Environmental chemistry, Environmental impact

## Abstract

Little attention has been given to the development of remediation strategies for soils polluted with mixture of pollution (metal(loid)s and organic compounds). The present study evaluates the effectiveness of different types of commercial iron nanoparticles (nanoscale zero valent iron (nZVI), bimetallic nZVI-Pd, and nano-magnetite (nFe_3_O_4_)), for the remediation of an industrial soil co-contaminated with Cr and PCBs. Soil samples were mixed with nZVI, nZVI-Pd, or nFe_3_O_4_ at doses selected according to their reactivity with PCBs, homogenized, saturated with water and incubated at controlled conditions for 15, 45 and 70 days. For each sampling time, PCBs and chromium were analyzed in aqueous and soil fractions. Cr(VI) and Cr leachability (TCLP test) were determined in the soil samples. The treatment with the three types of iron nanoparticles showed significant reduction in Cr concentration in aqueous extracts at the three sampling times (> 98%), compared to the control samples. The leachability of Cr in treated soil samples also decreased and was stable throughout the experiment. Results suggested that nZVI and nZVI-Pd immobilized Cr through adsorption of Cr(VI) on the shell and reduction to Cr(III). The mechanism of interaction of nFe_3_O_4_ and Cr(VI) included adsorption and reduction although its reducing character was lower than those of ZVI nanoparticles. PCBs significantly decreased in soil samples (up to 68%), after 15 days of treatment with the three types of nanoparticles. However, nFe_3_O_4_ evidenced reversible adsorption of PCBs after 45 days. In general, nZVI-Pd reduced PCB concentration in soil faster than nZVI. Control soils showed a similar reduction in PCBs concentration as those obtained with nZVI and nZVI-Pd after a longer time (45 days). This is likely due to natural bioremediation, although it was not effective for Cr remediation. Results suggest that the addition of nZVI or nZVI-Pd and pseudo-anaerobic conditions could be used for the recovery of soil co-contaminated with Cr and PCBs.

## Introduction

Soil pollution is a worldwide issue due to its impact on the degradation of ecosystem services provided by the soil, reducing food security and affecting human health^1^. The majority of pollutants have anthropogenic origins, such as industrial processes, mining, transportation, and land application of sewage sludge^2–4^. Metals and metalloids are the main contaminants found in European soils^4^. They are non-biodegradable as organic pollutants and they therefore persist in soil and sediment for extended periods of time after their introduction. Chromium is widely used in many industrial activities because of its corrosion-resistant properties. These include metallurgical processes, leather tanning, timber treatment, electroplating, and petroleum refining^5^. The most common forms of Cr found in soils are Cr(III), which occurs as insoluble oxide and hydroxide cations, and Cr(VI), which appears as oxyanion, is repelled by the negative charge of soil, as such, it remains in soil solution, available to plants and other organisms present in soils^5, 6^. Consequently, Cr(VI) is highly mobile and bioavailable, and it is considered 1000 times more toxic than Cr(III)^2, 3, 7^.

Together with metals, persistent organic pollutants (POPs) are organic chemical substances that pose a threat to the environment, because they are persistent, highly toxic, and bioaccumulative. POPs include some pesticides (e.g. DDT), unintentionally formed products (such as dioxins and furans) and industrial chemicals (such as polycyclic aromatic hydrocarbons, PAHs, and polychlorinated biphenyls, PCBs)^8, 9^. PCBs are a broad group of synthetic organic compounds included in the Stockholm Convention of 2001 as POPs due to their high potential for bioaccumulation and resistance to degradation. Owing to their properties, PCBs have many industrial applications, such as dielectric fluids in transformers and capacitors, hydraulic fluids, plasticizers in paints, copying papers, adhesives, sealants, formulations of lubricants, liquid seals and cutting oils. Moreover, PCBs can be unintentionally produced as by-products in a wide variety of chemical processes that contain chlorine and hydrocarbon sources^10^. The presence of PCBs in soil has been related to leakage from electrical transformers, application of wastes to land, emissions from waste incinerators, spills during transport, volatilization and deposition from surface waters, and leakage from inappropriate disposal in landfills^11^. There are about 209 possible congeners depending on the substitution of the chlorine atoms around the biphenyl molecule, but PCBs research has been mainly focused on a few number of representative congeners which largely contribute to the total amount found in the environment, such as PCB28, PCB52, PCB101, PCB138, PCB153 and PCB180.

According to the United States Environmental Protection Agency (USEPA), there are tens of thousands of polluted sites across the country, and a total of 1,335 of these are included on the Superfund National Priorities List^12^. In the EU, it is estimated that there are 2.8 million potentially contaminated sites, of which about 14% require urgent remediation^4, 13^. Currently, one of the priority objectives of the EU is to protect, conserve, and enhance the Union’s natural capital, and for that the EU is committed to sustainable land management, adequate soil protection, and the rehabilitation of contaminated sites (Decision No. 1386/2013/EU, EU Soil Thematic Strategy for 2030). In the last decades different methodologies have been applied to recover soils polluted with metal(loid)s or with organic pollutants, including bioremediation, phytoremediation, electrokinetic remediation, soil washing, stabilization and solidification^8, 14^. The efficiency of each technology depends on soil properties and pollutant characteristics as well as economic criteria and time^15^. In many cases metals and organic pollutants co-exist in the soil^16–21^. Particularly, co-contamination with Cr and PCBs can be due to effluents from different industries^21^. The remediation of co-contaminated soils is often more complicated due to the interactions among pollutants including changes of solubility, speciation and bioavailability, competition for the binding sites of adsorbents and inhibition of microbial metabolism which can conduct to synergistic and antagonistic effects on the remediation results^22^. In this regard, some researches have been focused on the remediation of co-contaminated soils with organic compounds and metal(loid)s including bioremediation, phytoremediation, treatment with agents and combined techniques such as electrokinetic-bioremediation^22–25^. Cao et al.^23^ got promising results for the remediation of soil and water samples artificially polluted with PCBs, Cu and Pb with zero valent iron and the biodegradable ligand EDDS. The exposure to PCBs, metals and metalloids poses a serious risk for both human health and the environment, thus the development of effective remediation techniques is very important and should be prioritized^26^.

Recently, nanotechnology has enabled the generation of new cost-effective and environmentally friendly remediation strategies, compared with traditional physico-chemical technologies^27–29^. Nanoparticles have a large specific surface area which contributes to increase rates of reaction with pollutants^27, 29–31^. Many different nanoparticles have been tested for remediation purposes, including carbon nanotubes, metal oxides and zeolites but nanoscale zero valent iron (nZVI) particles are the most widely used^28, 32^. Zero valent iron nanoparticles have a core–shell structure which is fundamental for interaction with different pollutants. The core consists of Fe^0^ and is responsible for the reduction processes, and the shell, formed of iron oxides and hydroxides (Fe^2+^ and Fe^3+^) as a result of spontaneous Fe^0^ corrosion, can have metal-like or ligand-like coordination properties and allows electron transfer^33–35^. Thus, applications of nZVI have focused on electron-donation and sorption and/or complexation properties. In this regard, nZVI has been found to be effective in the immobilization of metals and metalloids in water and soil samples^28–30, 33–44^. In addition, some researches have shown the potential of nZVI for degradation of organic pollutants in water such as halogenated aliphatic and aromatic compounds, pesticides, dyes, explosives and pharmaceutical compounds^32, 33, 45–50^. The use of bimetallic nZVI, obtained from the addition of small amounts of a transition metal (Ag, Cu, Ni, Pd or Pt), increases the reactivity of nZVI^29, 30^. Since Wang and Zhang^31^ concluded that nZVI and nZVI-Pd completely dechlorinated several chlorinated aliphatic compounds and a mixture of PCBs in aqueous media, other researches have confirmed their results^51–53^. The application of nZVI for PCBs degradation in soil samples has also been tested, although sometimes under more energetic experimental conditions, with higher doses compared to those applied in water. In addition, soil properties decisively affect the nanoparticle effectiveness for PCBs degradation. In this regard, Varanasi et al.^54^ found that the application of nZVI at 300 °C in air together with iron oxide and V_2_O_5_/TiO_2_ were good catalysts for remediating PCB contaminated soils. Sevcu et al.^55^ observed that different types of nZVI and ZVI at micro scale was highly efficient for PCBs degradation in water, but they had little degradation capacity in soil samples. Chen et al.^56^ found that nZVI and nZVI-Pd (especially the latter), were promising for the degradation of PCBs in soil samples when 3 g of soil was mixed with 1 g of nZVI, although soil properties decisively affected their effectiveness. nZVI and nZVI-Ni were effective for PCBs degradation in spiked sand but in historically contaminated soil, the effectiveness was limited (13–19%)^57^.

In recent years, interest has increased for the use of magnetic iron oxide nanoparticles such as nanomagnetite (nFe_3_O_4_), for the remediation of metal(loid) polluted waters. This is due to their high adsorption capacities and magnetic properties which enhance separation from the bulk medium by a magnetic force^58–67^. Although the available data regarding the application of nFe_3_O_4_ for remediation of organic pollutants is limited, several studies have shown good results as sorbent of PCBs^68, 69^. In this regard, Baragano et al.,^16^ applied this kind of nanoparticles to a soil contaminated with As and PAH, finding high yields of pollutant immobilization and reduction of soil toxicity. However, little data is available on the effectiveness of nFe_3_O_4_ for remediation of soil polluted with PCBs. Rybnikova et al.^57^ applied nFe_3_O_4_ as a catalyzer in a Fenton process together with an oxidizing agent, H_2_O_2_ or K_2_S_2_O_8_, and found degradation of PCBs in a spiked sand (> 69%), whereas in a historically polluted soil, the same conditions showed a limited degradation capacity (7–8%).

The application of nanoremediation strategies to soil polluted with a mixture of pollutants is scarce. In particular, there are few data available regarding the effectiveness of nanoremediation strategies for the simultaneous remediation of soils co-contaminated with metals and PCBs. In this context, the main objective of the present study was to evaluate the effectiveness of different types of commercial iron nanoparticles (nZVI, nZVI-Pd and nFe_3_O_4_) for the remediation of an industrial soil co-contaminated with Cr and PCBs. To the best of our knowledge, this is the first study which compares the effectiveness of different types of iron nanoparticles for the recovery of a soil with mixed pollution (Cr and PCBs).

## Materials and methods

### Reagents and standards

Ethyl acetate (EtAc) and n-hexane residue analysis grade, were purchased from Scharlau (Barcelona, Spain). A standard solution PCB-Mix containing PCB28, PCB52, PCB101, PCB138, PCB153 and PCB180 at a concentration of 10 µg/mL in iso-octane was purchased from Fluka (Seelze, Germany). Working mixture solutions of PCBs at 1 µg/mL were prepared in iso-octane. All standard solutions were stored at 4 °C prior to use. Mean concentration of soil PCBs was 2.3 mg/kg.

### Iron nanoparticles

Three types of commercial iron nanoparticles, nZVI, nZVI with Pd as catalyzer (nZVI-Pd) and nFe_3_O_4_ were included in the present study. Table 1 shows information about their manufacture and main characteristics. Zeta potential was determined with a Zetasizer nanoZ (Malvern Instruments Ltd.). Morphological analysis of the iron nanoparticles was performed by Scanning Electron Microscopy (SEM) using a JEOL JSM-7600F microscope. The X-ray diffraction (XRD) patterns were collected on a Bruker D8 Advance diffractometer with Cu kα1 radiation (1.54060 Å), and a diffraction angle (2θ) from 5° to 90° with step size 0.04°. The surface chemistry of the nanoparticles was analyzed by X-ray photoelectron spectrometry (XPS) using a PerkinElmer PHI 5400 spectrometer equipped with a Mg Kα excitation source (hν = 1253.6 eV) and a beam size with a diameter of 1 mm following the methodology previously described^38^. Attenuated total reflectance-Fourier transform infrared spectroscopy (ATR-FTIR) was performed using a Shimadzu IRAffinity-1S apparatus with a diamond ATR accessory between 400 and 4000 cm^−1^, 64 scans with a resolution of 8 cm^−1^ and a mirror velocity of 0.6329 cm/s^38^.

**Table 1:** Characteristics of the iron nanoparticles used in the study.

Characteristic	nZVI	nZVI-Pd	nFe_3_O_4_
Producer	NanoIron (Czech Republic)	NanoIron (Czech Republic)	IoliTec Nanomaterials (Germany)
Physical state	Aqueous suspension (80% water)	Aqueous suspension (80% water)	Solid
Composition	14–18% Fe(0) y 2–6% Fe_3_O_4_	14–18% Fe(0) y 2–6% Fe_3_O_4_, Pd 0.1%	Iron(II,III) oxide > 98%
Organic stabilizer	3% polyacrylic acid	3% polyacrylic acid	–
Mean diameter (nm)	50	50	20–30
Specific surface area (m^2^/g)	> 25	> 25	90
Zeta potential (mV)	− 31.9	− 21.2	− 10.3

### Soil

Soil samples from an industrial area located in Oviedo (North of Spain) historically polluted with PCBs were collected from the surface layer (0–30 cm), air-dried and sieved (< 2 mm). Physico-chemical soil properties were determined using the Spanish official methodology for soil analysis^70^ and are shown in Table 2. Briefly, pH and electrical conductivity (EC) were measured in a 1:2.5 soil-to-water ratio; available phosphorous was determined after extraction with sodium bicarbonate at pH 8.5; organic matter and total nitrogen were analyzed by the Walkley–Black and the Kjeldahl methods, respectively; available Na, Mg, Ca and K were extracted with ammonium acetate 0.1 N and quantified using a flame atomic absorption spectrometer (AA240FS, Varian); the particle size distribution of the soil was determined by the Pipette method; the concentration of Cd, Cr, Cu, Ni, Pb, Pd and Zn was measured by acid digestion with a mixture of HNO_3_ (6 mL, 69%) and HCl (2 mL, 37%) in a microwave reaction system (Multiwave Go, Anton Paar GmbH), followed by the analysis of the metals in the digestion extracts by flame atomic absorption spectrometry. The limits of quantitation were in the range of 0.1 to 1.0 µg/g. The results of blank analysis were always below the detection limit, and soil reference material (SQC001, Sigma-Aldrich) recoveries were within the certified value (88–104%).

**Table 2:** Physico-chemical soil properties and concentration of PCBs.

Parameter	Soil
pH	6.29
EC (dS/m)	2.7
N (%)	0.06
OM (%)	0.81
Ca (mg/kg)	594
Mg (mg/kg)	90
Na (mg/kg)	31
K (mg/kg)	155
Cd (mg/kg)	< LD
Cr (mg/kg)	214
Cr(VI) (mg/kg)	65
Cu (mg/kg)	9.1
Ni (mg/kg)	< LD
Pb (mg/kg)	10
Pd (mg/kg)	< LD
Zn (mg/kg)	30
Sand (%)	64
Silt (%)	25
Clay (%)	11
PCB28 (*tri*-CB) (ng/g)	33.9
PCB52 (*tetra*-CB) (ng/g)	193
PCB101 (*penta*-CB) (ng/g)	512
PCB138 (*hexa*-CB) (ng/g)	719
PCB153 (*hexa*-CB) (ng/g)	531
PCB180 (*hepta*-CB) (ng/g)	292

The mean concentration of Cd, Cu, Ni, Pb and Zn were below the current Regional Screening Levels for industrial uses in Asturias^71^ (Table 1). In contrast, the concentration of Cr(VI) was above the allowed value (2–50 mg/kg, depending on the land uses). Regarding the PCBs, the sum of the analyzed PCBs exceeded almost three times the maximum level allowed for industrial use in Spanish legsilation^72^. Thus, given these concentrations of Cr(VI) and PCBs and according to the legislation limits,^71, 72^ this soil has a contamination level which presents ecological and/or health risks, and needs to be remediated.

After nanoparticle treatment, Cr availability in soil samples was evaluated using the TCLP (Toxicity Characteristic Leaching Procedure) test (USEPA 1311). In brief, 20 mL of sodium acetate buffer (0.1 M, pH 4.93 ± 0.05) was mixed with 1 g of soil, shaken overnight and the concentration of Cr in the extract was quantified by flame atomic absorption spectrometry.

### Batch experiment

Fifteen grams of soil sample was weighed in a plastic tube (50 mL Falcon) and mixed with the iron nanoparticles at 10% (20 g Fe/kg) dose for nZVI and nZVI-Pd, and 5% for nFe_3_O_4_ (36.2 g Fe/kg). The doses were selected based on previous assays. Lower doses of iron nanoparticles did not induce significant degradation of PCBs in this soil (data not published). Then, the mixture was homogenized, and 35 mL of water was added to completely fill the tube and minimize the presence of oxygen. Tubes were capped. These pseudo-anaerobic conditions were chosen to prevent nanoparticle oxidation. In order to evaluate the potential bioremediation process in soil, control samples without iron nanoparticles were also included. Tubes were incubated at 28 °C in a climatic chamber, at darkness for 15, 45 and 70 days. During the first 24 h, the tubes were depressurized several times. In each sampling time, tubes were centrifuged, and supernatant was collected with a Pasteur pipette, filtered and stored at 4 °C until PCBs and Cr analysis was carried out. Soil was air-dried for the analysis of PCBs, Cr(VI) and Cr and Pd-leachability. The leachability tests were performed according to the TCLP test. Experiments were carried out in triplicate.

### Chromium speciation

The concentration of Cr(VI) in soil samples was determined using ion chromatography with an UV–VIS detector at 365 nm according to the method described by Phesatcha et al.^73^. Briefly, 0.1 g of soil sample was mixed with nitric acid (5 mL, 50 mM), 10 mL eluent stock (2 mM pyridine-2,6-dicarboxylic acid, 2 mM disodium hydrogen phosphate anhydrous, 10 mM sodium iodide, 50 mM ammonium acetate and 2.8 mM lithium hydroxide) and 10 μL nitric acid (69%). The mixture was heated for 30 min, cooled to room temperature and filtered before injection in the chromatograph.

### PCBs extraction in soil aqueous extract

PCBs in aqueous extract were extracted by liquid–liquid extraction. Soil leachate (10 mL) was shaken with hexane (2 × 10 mL) and EtAc (2 × 10 mL) using a separatory funnel. After shaking, the combined organic extracts were concentrated to 1 mL using a Genevac EZ-2 evaporator (NET Interlab, Spain). A small spatula-tip full of anhydrous sodium sulfate was then added to the organic extract to remove any water it may contain prior to its GC–MS analysis.

### PCBs extraction in soil samples

For PCBs determination in soil samples, 1 g of soil was placed in a 20 mL glass column with a cellulose paper filter at the bottom and 1 g of Florisil. The columns were closed with one-way stopcocks before adding the extraction solvent (4 mL of EtAc). They were placed in an ultrasonic water bath at room temperature for a 15 min sonication cycle. They were then placed on a multiport vacuum manifold and the eluates were collected in conical tubes. This procedure was repeated with another 4 mL of the extraction solvent. The combined extracts were adjusted to 3 mL prior to analysis by GC–MS.

### GC–MS analysis

The analysis was performed with an Agilent 6890 gas chromatograph equipped with an automatic injector, HP 7683, coupled to a quadrupole mass spectrometer model 5977. Separations were carried out using a fused silica capillary column ZB-5MS, 5% phenyl polysiloxane as nonpolar stationary phase (30 m × 0.25 mm i.d. and 0.25 µm film thickness), from Phenomenex (Torrance, CA).

Helium (purity 99.995%) was used as carrier gas at a constant flow rate of 1 mL/min. Injections were carried out with 2 µL of extract in the pulsed splitless mode with the injection port at 285 °C, pulsed pressure 45 psi for 1.5 min, with the splitless injector purge valve activated 1.5 min after sample injection, in a glass liner with deactivated glass wool. The column temperature was initially set at 80 °C (held for 0.5 min), increased at 20 °C/min to 280 °C (held for 4 min). The total time of the analysis was 14.7 min.

A stock solution of PCBs was made up at 2 µg/mL level in EtAc and the standard solutions were stored in glass flasks at 4 °C. The quantification of the PCB isomers was based on their relative response to external standards. The linear range was established by a five-point calibration curve in the range 12.5–250 ng/mL.

The target and qualifier abundances were determined by injecting standards under the same chromatographic conditions, using full-scan with the mass/charge ratio ranging from 100 to 800 m/z. The chromatographic method was divided in five time segments. Table S1 lists the mass spectrometry parameters of the PCBs for the GC–MS method. Retention times must be within ± 0.2 min of the expected time and qualifier-to-target ratios within a 20% range for positive confirmation.

### Statistical analysis

Data were statistically analyzed using the IBM SPSS package for Windows, release 19.0.0.1. The normal distribution of all variables was checked by a Kolmogorov–Smirnov test. Difference among treatments were determined by one-way analysis of variance at significance level of p < 0.05, followed by a Tukey post-hoc test.

## Results and discussion

### Characterization of iron nanoparticles

**Figure 1:**
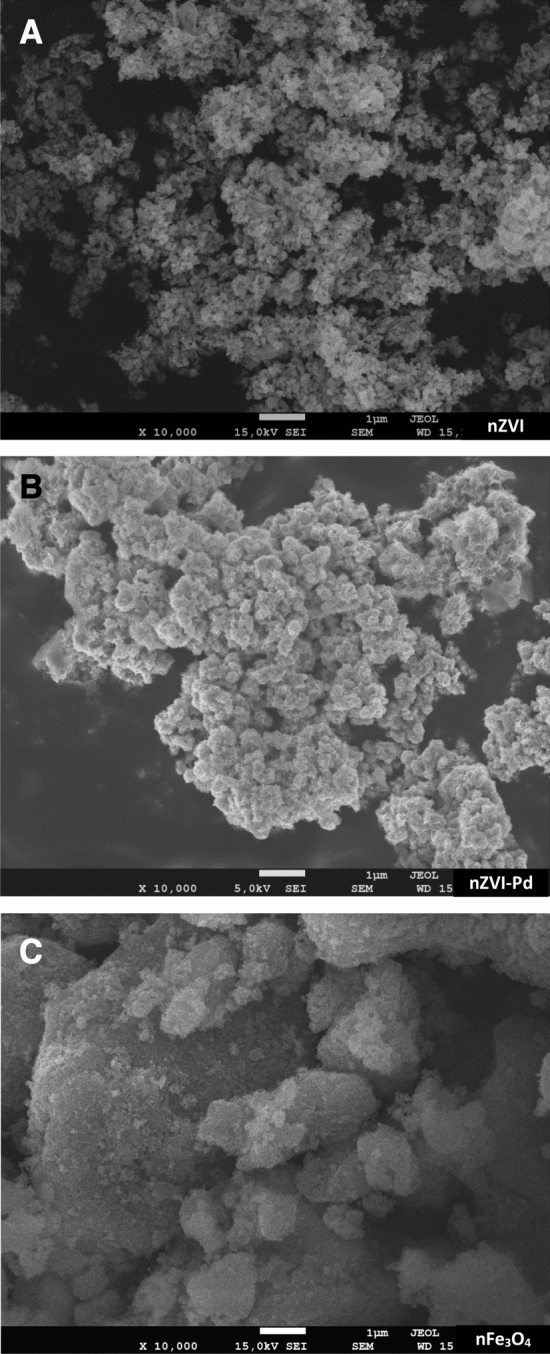
SEM imagines of nZVI (**A**), nZVI-Pd (**B**) and nFe_3_O_4_ (**C**).

SEM images of the three iron nanoparticles are shown in Fig. 1. Regarding nZVI, most of the nanoparticles are spherical with a regular size and forming chain like aggregates. Bimetallic nanoparticles showed a similar surface topography to nZVI formed by spheres and chain-like aggregates. Magnetite nanoparticles were made of spherical particles with irregular sizes.

**Figure 2:**
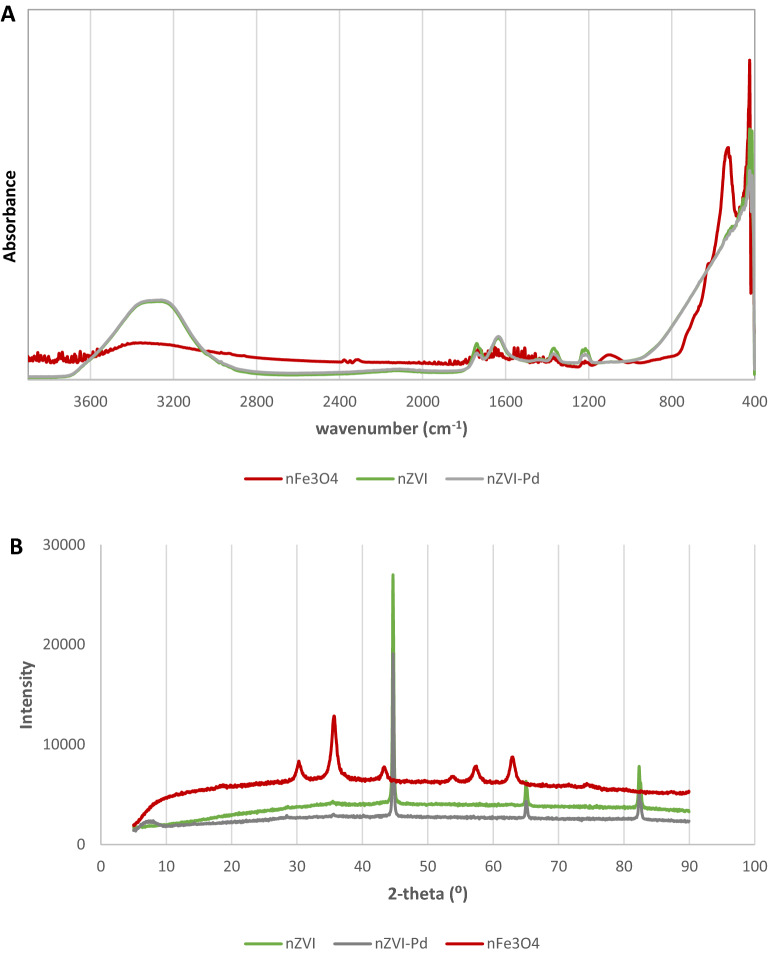
FTIR spectra (**A**) and powder XRD pattern (**B**) of nZVI, and nZVI-Pd nFe_3_O_4_.

Figure 2 shows the FTIR spectra and the powder XRD patterns of the three types of iron nanoparticles. The FTIR spectra of nZVI and nZVI-Pd are similar, with an intense absorption peak near 3400 cm^−1^ related to O−H stretch from water. The band at 1600 cm^−1^ can be due to O–H–O scissors-bending or to the –C=O stretch of the carboxylic acid of the stabilizer (sodium salt of polyacrylic acid, Table 1)^74^. The bands between 570 and 400 cm^−1^, characteristic of the Fe–O bond, confirm the presence of iron oxides^38, 65, 75^. FTIR spectrum of nFe_3_O_4_ showed two strong absorption bands at 540 and 430 cm^−1^, which can be assigned to the Fe–O stretching mode of the tetrahedral and octahedral sites^76^.

As can be noted, nZVI and nZVI-Pd exhibit rather similar XRD patterns with diffraction peaks at 2θ = 44.64°, 65.01° and 82.32°, characteristic of α-Fe^0^ (ICDD 04-013-5208, 04-006-3633), and a weaker peak at 35.48° which can be attributed to iron oxide (ICDD 00-003-0863, 01-084-2782). These results verified that nZVI has a core–shell structure, a core of Fe^0^ and a thin shell constituted by iron oxides. Regarding the XRD pattern of nFe_3_O_4_, the diffraction peaks (2θ = 30.29°, 35.69°, 43.31°, 53.79°, 57.33°, 63.06° and 74.33°) are in good agreement with the standard data of magnetite (ICDD, 04-013-9808) and no other peaks are detected, indicating that the nanoparticle are pure phase Fe_3_O_4_.

**Figure 3:**
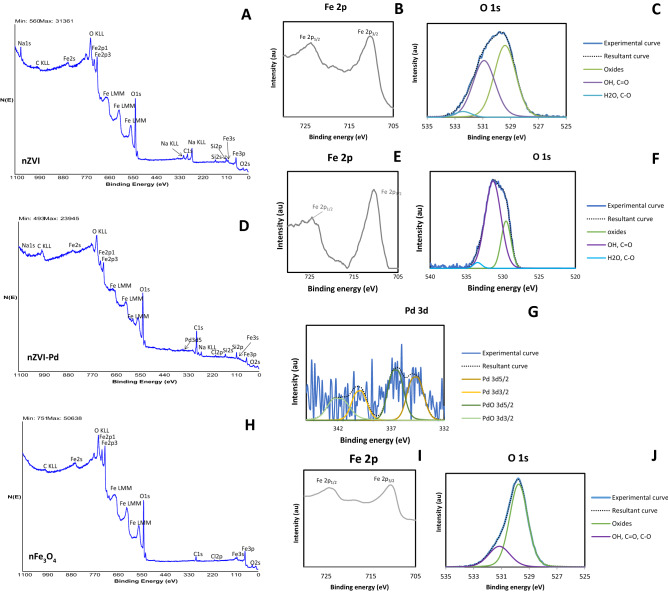
Wide scan XPS spectra for nZVI (**A**), corresponding high resolution spectrum of Fe 2p (**B**) and O 1s (**C**); wide scan XPS spectra for nZVI-Pd (**D**), corresponding high resolution spectrum of Fe 2p (**E**), O 1s (**F**) and Pd 3d (**G**); wide scan XPS spectra for nFe_3_O_4_ (**H**), high resolution spectrum of Fe 2p (**I**) and O 1s (**J**).

Figure 3 collects the XPS spectra obtained for the three nanoparticles. nZVI and nZVI-Pd showed a similar pattern, being iron and oxygen quantitatively the most important elements; sodium and carbon come from the organic stabilizer (Table 1) and Si may be an impurity of the nanoparticles as concluded in a previous study^38^. The spectra of O 1*s* region (Fig. 3C,F) included oxides (529 eV), hydroxides (531 eV) and chemically or physically adsorbed water (532 eV), characteristics of the iron oxide from the nZVI surface^34^. On the basis of the binding energy and the form of the Fe 2*p* peak of nZVI (Fig. 3B) we can concluded that αFe_2_O_3_ predominates on the surface of nZVI. In the case of nZVI-Pd, αFe_2_O_3_ and FeOOH were found on the surface. No metallic iron was identified in either nZVI or nZVI-Pd probably due to XPS technique analyzes the surface of the nanoparticles and metallic iron is present on the core of nZVI^38, 77^. A low proportion of Pd was also detected on bimetallic nanoparticle surface (Fig. 3G). The Pd 3d spectrum showed Pd 3d_5/2_ signals at 334.7 eV and 336.5 eV, characteristic of metallic Pd and PdO, respectively^77–79^. Huang et al.^77^ also detected metallic Pd and Pd^2+^ on nZVI-Pd surface. Regarding the nFe_3_O_4_, the Fe 2p_3/2_ spectrum (Fig. 3I) showed a binding energy of 711 eV and a satellite peak located at 719 eV, characteristic of Fe^3+^. This observation, together with the narrow shape of the main Fe 2p_3/2_ peak, suggests that iron is present mainly as maghemite (γFe_2_O_3_)^80^, which can be formed through the oxidation of magnetite. The region of O 1*s* showed that oxygen is present mainly as oxide and hydroxide at the magnetite surface. The magnitude of zeta potential indicates the colloidal stability of the nanoparticles; values more positive than 30 mV or more negative than − 30 mV, are considered stable, with maximum instability (i.e., aggregation) occurring at a zeta potential of 0^30^. In the present study, the colloidal stability in decreasing order was nZVI > nZVI-Pd > nFe_3_O_4_ (Table 1).

### Method validation of PCBs analysis

After optimization, the developed method was evaluated in terms of linearity, accuracy precision and detection limits before it was used to determine the concentrations of PCBs at the different times in the soil samples. Detailed information about its validation is provided in the Supplementary section.

### Cr in soil aqueous fraction

**Figure 4:**
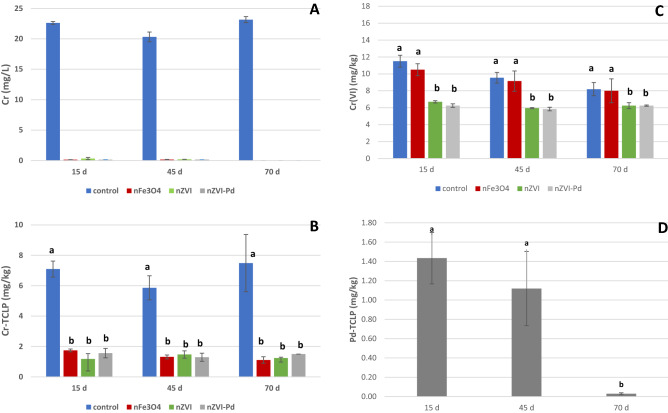
(**A**) Mean concentration of Cr (mg/L) in the aqueous extracts at the different sampling times. (**B**) Mean concentration of Cr (mg/kg) in TCLP extracts at the different sampling times. (**C**) Mean concentration of Cr(VI) (mg/kg) in soil samples at the different sampling times. For each sampling time, bars with the same letter do not differ significantly (p < 0.05). (**D**) Mean concentration of Pd (mg/kg) in TCLP extracts at the different sampling times for nZVI-Pd treated soils. Bars with the same letter do not differ significantly (p < 0.05).

The addition of nZVI, nZVI-Pd and nFe_3_O_4_ significantly reduced Cr concentration in the extracts at the three sampling times (Fig. 4A). In contrast, control samples showed a higher Cr concentration (close to 22 mg/L) which was similar throughout the experiment. Most of the Cr present in aqueous fraction is likely Cr(VI) since Cr(III) it is almost completely precipitated at pH 5.5 or higher^2, 3^. No differences were detected among the nanoparticle treatments and after 15 days of interaction, the Cr concentration in aqueous extract of treated samples was below 0.4 mg/L, after 45 days it was below 0.2 mg/L and after 70 days below the quantitation limit for the three iron nanoparticles (0.06 mg/L). In this regard, Wang et al.^81^ did not find Cr in the aqueous phase after 72 h of interaction between Cr-polluted soil and nZVI stabilized with carboxymethyl cellulose (CMC) in aqueous medium.

### Cr availability in soil samples

Cr availability in soil samples was evaluated by considering the potential Cr leachability using the TCLP test (Fig. 4B). The addition of iron nanoparticles significantly reduced the TCLP-Cr which agrees with the previous results in water extracts. In this regard, TCLP-Cr in control samples was between 5.9 and 7.5 mg/kg, significantly higher than those found in treated soils which ranged from 1.1 to 1.7 mg/kg. No significant differences were observed in Cr immobilization among the three types of iron nanoparticles. In relation to the sampling times, no differences were found among them for each treatment, for both the treated and control soils. In summary, the addition of nZVI, nZVI-Pd or nFe_3_O_4_ to Cr-polluted soil significantly reduced the leachability of Cr in soil and the immobilization was stable for at least 70 days under the experimental conditions.

### Cr(VI) in soil samples

The concentration of Cr(VI) in soil samples collected after separation from the aqueous phase is shown in Fig. 4C. The soil samples treated with nZVI and nZVI-Pd showed similar concentrations of Cr(VI) at the three sampling times (15, 45 and 70 days), with a mean value close to 6 mg/kg, significantly lower than the initial concentration (65 mg/kg). However, the TCLP values for these soil samples were below 1.6 mg/kg (likely as Cr(VI)). These results demonstrate that Cr was immobilized as Cr(III) and Cr(VI). Thus, the interaction mechanism between nZVI and Cr(VI) in the soil samples implied the combined process of reduction to Cr(III) and adsorption of Cr(VI) on the shell of the nanoparticle as other authors have concluded^81–84^. In this regard, Wang et al.^84^ applied CMC-nZVI to a Cr(VI)-polluted soil and they concluded that the Cr(VI) was initially absorbed into the shell of nZVI, then, most of it was gradually reduced to Cr(III) whereas iron and chromium oxidation products (Fe(III) and Cr(III) oxides/hydroxides) as well as the products of the hydrolyzation of the CMC covered the nZVI, limiting contact of the Cr(VI) with the reductant Fe(0). According to the reduction potential (E^0^ (Fe^2+^) = − 0.41 V, E^0^ (Cr^6+^) = 1.36 V), the reduction of Cr(VI) to Cr(III) is thermodynamically favorable forming Cr(OH)_3_ and Cr–Fe (oxy)hydroxide^34, 82, 83, 85, 86^. The same interaction mechanism exists for nZVI and nZVI-Pd; the main difference between both iron nanoparticles is that Pd acts as a catalyst increasing the reactivity of the nanoparticles. In the present study, no significant differences were observed for Cr(VI) immobilization between nZVI and nZVI-Pd, probably due to the high dose of nanoparticles and the long contact time used in the assay. Previous experiments have extensively shown high effectiveness of nZVI for Cr immobilization in soil samples^39, 40, 81, 84, 87–90^.

Soil samples treated with nFe_3_O_4_ showed similar concentration of Cr(VI) as the controls and they were higher than those found in nZVI and nZVI-Pd treatments for all the sampling times. However, it should be noted that the control samples exhibited much of the mobile chromium in the aqueous fraction (Fig. 4A). No significant changes among sampling times were detected. The concentrations of Cr(VI) found in soil samples treated with nFe_3_O_4_ were in the range of 8.5 and 10.5 mg/kg, higher than Cr-TCLP (Fig. 4B). Thus, reduction and adsorption were employed for Cr(VI) removal. In this case, Cr(VI) reduction was favored by the presence of Fe(II) from the magnetite (Fe(II)Fe(III)_2_O_4_)^91–93^. However, the reducing character of nFe_3_O_4_ was lower than nZVI particles which contains Fe^0^ and Fe^2+^ as reducing agents. The application of nFe_3_O_4_ with different modifications has been effectively used as an adsorbent for the decontamination of Cr-polluted waters or inert materials like sand^65, 92, 94^. However, to the best of our knowledge, little data is available on the effectiveness of nano-magnetite for Cr immobilization in polluted soils. Similar results were found in the three sampling times.

Control soil samples presented mean values lower than that found in the original soil. This may be because most of the Cr present in aqueous phase was probably Cr(VI), the most mobile form of Cr. The decrease of the concentration of Cr(VI) over time can be due to its reduction to Cr(III) by abiotic and biotic processes^5, 95^. The presence of Fe(II), reduced sulfur compounds and organic matter (humic and fulvic acids) can be a source of electrons for reducing Cr(VI)^5, 96^.

### Pd in soil samples

Given that Pd is present in nZVI-Pd formulation, this element was analyzed in the soil samples. There is little information on how soil treatment with nZVI-Pd particles impacts Pd mobility in this matrix. In the present study, neither the untreated polluted soil nor the samples treated with nZVI and nFe_3_O_4,_ showed Pd. Nevertheless, the addition of nZVI-Pd under the experimental conditions induced the incorporation of this element into soil, with a mean value of 150 ± 38 mg/kg. Currently, Pd is not included in European legislation on polluted soils. Palladium availability, as evaluated using the TCLP test, revealed mean values of 1.43, 1.11, and 0.03 mg/kg after 15, 45 and 70 days of treatment, respectively (Fig. 4D). Thus, a low percentage of the total Pd was available (a maximum of 0.95% at the first sampling time), and this parameter decreased significantly over time (p < 0.05).

### PCBs in soil aqueous fraction and soil

**Figure 5:**
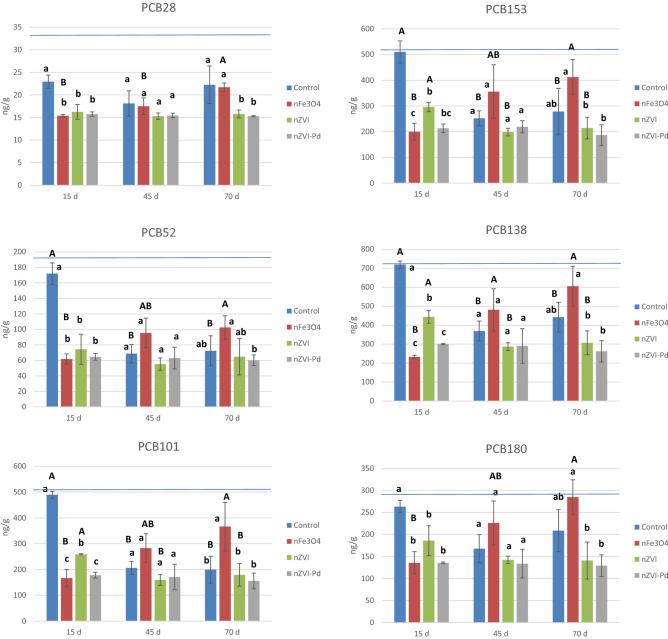
Mean concentration of PCBs (ng/g) in soil samples at the different sampling times. Bars with the same letter do not differ significantly (p < 0.05); lower letters compare among treatments for the same sampling time; upper letters compare among sampling times for the same treatment. Blue horizontal line indicates the initial PCB concentration.

PCBs were not detected in aqueous fraction for any of the treatments at any sampling time, probably due to their high hydrophobicity. The PCBs mean concentration in the soil samples at the different sampling times is shown in Fig. 5. After 15 days of interaction between polluted soil and iron nanoparticles, a significant decrease of PCBs was observed for all the studied PCBs. The three types of iron nanoparticles significantly reduced the PCBs concentration under the experimental conditions after 15 days; nFe_3_O_4_ and nZVI-Pd showed similar reduction results, and they were significantly more effective than nZVI for PCB101, PCB153 and PCB138. The PCB28 was the least relevant quantitatively, and the reduction rate was also the lowest (close to 30%). The decrease of PCB52 was between 57 and 64%. For the PCB101, decreases of 66%, 47% and 64% were observed for nFe_3_O_4_, nZVI and nZVI-Pd, respectively. Percentages of decrease of 60%, 42% and 58% for PCB153, of 68%, 38% and 58% for PCB138, of 48%, 29% and 48% for PCB180 were obtained for nFe_3_O_4_, nZVI and nZVI-Pd, respectively after 15 days of interaction. Thus, the present results show the capacity of magnetite nanoparticles to reduce PCBs content in soil samples. However, the soil samples treated with nFe_3_O_4_ over a longer time period, 45 and 70 days, showed an increase in PCB content. We hypothesize that magnetite retains the PCBs by adsorption, but the interaction is reversible, and the PCBs are released over time. Thus, the PCBs concentration in soil samples treated with nFe_3_O_4_ was higher after 70 days of contact than those collected after 45 and 15 days. Thus, further studies are necessary to determine the optimum conditions of contact time, so as to evaluate the effectiveness of their use for reducing PCBs availability in soil, as well as the potential regeneration and reusability of the nFe_3_O_4_. In addition, due to the magnetic properties of nFe_3_O_4,_ they could be easily separated from the soil under a magnetic field, regenerated and reused several times^67^.

In the 15-day sampling, the bimetallic nanoparticles proved to be more effective and reduced PCB concentration faster than nZVI, especially in the case of PCB101, PCB153 and PCB 138. In the later samplings (45 and 70 days), no significant differences were found in the concentration of PCBs between soils treated with nZVI and those treated with nZVI-Pd. According to previous studies, ZVI nanoparticles degrade PCBs by reductive dechlorination: the PCB molecule accepts the electrons from Fe^0^ oxidation and is reduced, replacing the Cl atoms with H atoms^53, 56, 97^. He et al.^97^ concluded that the removal efficiency of the PCB101 in spiked soils improved with increasing concentration of bimetallic ZVI-Pd nanoparticles (both Fe and Pd dosage). The authors found an increase in the removal percentage from 20.3% at dose of 0.005% of Pd to 57.8% at 0.1% of Pd. This is probably due to two main reasons: i) the galvanic effects of the Fe-Pd system, in which Fe acts as anode and Pd as cathode, and the electrons released from Fe contribute to form Cl^-^; ii) Pd can strongly bond to Cl thereby accelerating the dissociation of chlorinated hydrocarbons; the H_2_ (g) produced during the iron oxidation is adsorbed on Pd and dissociated into atomic H, one of the strongest reductants for the dechlorination reactions^97–100^.

The comparison over time showed that the PCB concentrations did not change in soils treated with nZVI-Pd, no significant degradation was observed after 45 and 70 days. In those treated with nZVI, only PCB101, PCB153 and PCB138 significantly decreased after 45 days, however no more reductions were observed at longer time. The decrease in effectiveness of nZVI and nZVI-Pd over time is probably due to two main reasons. Firstly, the reducing power of ZVI decreases with time, due to oxidation of Fe(0) and aggregation of ZVI nanoparticles in the soil matrix, leading to a decrease of nanoparticle reactivity^99^. Although both nanoparticles contain an organic stabilizer to prevent the aggregation (Table 1), soil is a very complex matrix, and many different interactions can occur. Secondly, the different availability of the PCBs in soil matrix, i.e., the degraded PCBs were likely in the most available fraction of the soil and those most recalcitrant PCBs were not available to react with ZVI nanoparticles. PCBs can be strongly adsorbed to soil particles, especially organic matter which makes their interaction with nanoparticles difficult. In this regard, Varanasi et al.^54^ effectively reduced the concentration of PCBs in a soil after treatment with nZVI together with a nitrogen stream at 300 `C to allow the desorption of PCBs from the soil. In addition, as the soil is also polluted with Cr, this can induce competitive phenomena among pollutants to react with ZVI nanoparticles.

Control samples suffered a bioremediation process, showing the higher decrease of PCBs at 45 days remaining stable up to 70 days. The best results were observed for the PCB52, PCB101, PCB153 and PCB138, with removal rates between 49 and 60%. These reductions are likely associated with anaerobic biodegradation processes because of the pseudo-anaerobic conditions. Other authors have observed the microbial degradation of highly chlorinated PCB congeners under anaerobic conditions^48, 101^. Consequently, the concentrations of PCBs in control and treated samples were similar after 45 days. Thus, the application of nZVI-Pd and nZVI reduced the concentration of PCBs in soil in a similar range to those obtained by the bioremediation process in control samples but in less time. Similar PCBs concentration were found at 45 and 70 days; thus, no biodegradation processes occurred after 45 days of interaction at the experimental conditions. As previously explained, this can be due to biodegradation occurs with the most available fraction of PCBs, and the less available fraction is not easily accessible for microbiota. Thus, the PCBs availability could be the limiting factor for both, nanoremediation and bioremediation of PCBs in the polluted soil. Soil properties and degree of pollution would affect the remediation efficiency. According to the present results, bioremediation would be feasible for soil polluted exclusively with PCBs but not when soil also includes metals such as Cr.

## Conclusions

The addition of nZVI, nZVI-Pd or nFe_3_O_4_ to a soil co-contaminated with Cr and PCBs significantly reduced the leachability of Cr in soil and the immobilization was stable for at least 70 days under the experimental conditions. The nZVI and nZVI-Pd showed higher effectiveness for the reduction of Cr(VI) to Cr(III) compared to that of nFe_3_O_4_. After 15 days of interaction between soil-nanoparticles, the PCBs concentration significantly decreased in soils treated for the three types of iron nanoparticle. Magnetite nanoparticles exhibited a reversible process for PCBs adsorption. Soils treated with nZVI and nZVI-Pd showed a similar PCB degradation rate at 45 days of treatment. The latter approach required less time to degrade these compounds and was more effective even at 15 days; however, the use of nZVI-Pd implies the incorporation of Pd into the soil, although we observed that the available content was lower 1% of the total and it decreased over time. Due to bioremediation processes, the control soils showed a reduction in PCBs concentration in the 45-day sampling time, reaching similar values to those found in soils treated with nZVI and nZVI-Pd. In this regard, bioremediation would be feasible for soil polluted exclusively with PCBs but not when soil also includes metals such as Cr. Thus, nZVI based nanoparticles evidence a moderate efficacy for the remediation of PCB-polluted soils. In contrast, both nZVI nanoparticles exhibited successful results for the immobilization of Cr in soils. The results suggest that the addition of nZVI or nZVI-Pd and pseudo-anaerobic conditions could be used for the recovery of soils co-contaminated with Cr and PCBs.

## Supplementary Information

Supplementary Information.
