# Supplementary material for: Iron nanoparticles to recover a co-contaminated soil with Cr and PCBs
Source: Sci Rep. 2022 Mar 3;12:3541. doi: 10.1038/s41598-022-07558-w (PMC8894337; doi:10.1038/s41598-022-07558-w)
Supplement: Supplementary file 1 — Supplementary Information. [file 41598_2022_7558_MOESM1_ESM.docx]

**Iron nanoparticles to recover a co-contaminated soil with Cr and PCBs**

M. Gil-Díaz^1*^, R.A. Pérez^2^, J. Alonso^1^, E. Miguel^2^, S. Diez-Pascual^1^, M.C. Lobo^1^

^1^Dpto. Investigación Agroambiental, IMIDRA, Finca “El Encín”, A-2, km 38.2, Alcalá de Henares, 28805 Madrid, Spain

^2^Dpto. Medio Ambiente y Agronomía, INIA-CSIC, A-6, km 7, 28040 Madrid, Spain

^*^Corresponding author: M. Gil-Díaz

Phone 00 34 91 8879475

E-mail: [mar.gil.diaz@madrid.org](mailto:mar.gil.diaz@madrid.org)

**Supplementary material**

***Method validation of PCBs analysis***

After optimization, the developed method was evaluated in terms of linearity, accuracy precision and detection limits before it was used to determine the concentrations of PCBs at the different times in the soil samples. The linearity of the method was evaluated by injecting five standard solutions prepared at 5 to 100 ng/mL levels for all the studied compounds. A good linearity was obtained with correlation coefficients equal to or greater than 0.999 for all the PCBs studied.

The accuracy of the method was evaluated by performing the recovery of target analytes from soil samples spiked with standard solutions at two levels of concentration, 90 and 300 ng/g. Good recoveries (ranging from 97 to 106%) were obtained for the spiking levels assayed (Table 4). The repeatability was evaluated by analyzing a standard solution at 30 ng/mL. The sample was injected 8 times with an automatic injector and the relative standard deviations (RSD) obtained for peak areas were < 3%. Laboratory reproducibility of the chromatographic determination was evaluated on different days in two consecutive weeks and it was found to be lower than 13% for all of the isomers, expressed as RSD.

Limits of detection (LODs) and quantification (LOQs) were defined as the minimum amount of target analyte that produces a chromatogram peak with a signal-to-noise ratio of three and ten times the baseline noise, respectively. Therefore, LODs for soil samples were LODs ranged from 1.4 to 3.2 ng/g weight soil. The LOQ was calculated as 10 times the standard deviation of the results of the replicate analysis used to determine LOD and the values ranged from 4.2 to 9.6 ng/g (Table S2). Matrix effect was evaluated by preparing two sets of standard solutions ranging from 12.5 to 250 ng/mL, one set of standards was solvent-based and the other was prepared by spiking blank soil extracts. The slopes obtained from plotting five concentration levels against peak area, were compared using linear regression analysis, and no significant increase of the chromatographic response for PCBs was observed. Therefore, the chromatographic response of target analytes was not affected by the presence of matrix components and the quantifications were done using standard solutions in solvent.

**Table S1.** Retention time (t_R_), time segment (TS) retention time window (RTW), target (T), qualifier ions (Q1 and Q2) of the PCBs analyzed.

| Compound | t_R_  (min) | TS | RTW (min) | T  (m/z) | Q1  (m/z) | Q2  (m/z) |
| --- | --- | --- | --- | --- | --- | --- |
| PCB-28 | 8.4 | 1 | 7-9.4 | 256 | 258 | 186 |
| PCB-52 | 8.7 | 1 | 7-9.4 | 292 | 290 | 220 |
| PCB-101 | 9.6 | 2 | 9.4-9.9 | 326 | 328 | 254 |
| PCB-153 | 10.4 | 3 | 9.9-10.55 | 360 | 362 | 290 |
| PCB-138 | 10.7 | 4 | 10.55-11.0 | 360 | 362 | 290 |
| PCB-180 | 11.3 | 5 | 11.0-14.7 | 324 | 394 | 396 |

**Table S2.** Recoveries and relative standard deviations (RSD (n = 3, %, in parenthesis) obtained for the PCB isomers.

| Compound | Recovery (%) | |  | LOD | LOQ | |
| --- | --- | --- | --- | --- | --- | --- |
|  | 300 ng/g | 90 ng/g |  | (ng/g) | | (ng/g) |
| PCB-28 | 105.5 (3.3) | 96.5 (0.1) |  | 1.4 | | 4.2 |
| PCB-52 | 101.0 (1.0) | 100.6 (0.8) |  | 1.0 | | 2.9 |
| PCB-101 | 101.7 (2.0) | 102.9 (2.2) |  | 1.8 | | 5.4 |
| PCB-153 | 104.1 (0.1) | 105.3 (1.4) |  | 1.6 | | 4.7 |
| PCB-138 | 103.2 (0.2) | 104.9 (2.4) |  | 2.3 | | 6.8 |
| PCB-180 | 102.2 (0.1) | 102.1 (1.2) |  | 3.2 | | 9.6 |
